# Social Anxiety and Smartphone Addiction in Chinese University Students: A Moderated Mediation Model of Rumination and Gender

**DOI:** 10.3390/healthcare13080862

**Published:** 2025-04-09

**Authors:** Xuan Liu, Siti Mastura Binti Baharudin

**Affiliations:** School of Education Studies, Universiti Sains Malaysia, Penang 11800, Malaysia; xuan0924@student.usm.my

**Keywords:** university students, social anxiety, smartphone addiction, rumination, gender

## Abstract

**Background:** Research has shown that social anxiety can trigger smartphone addiction, but relatively few studies have explored the specific mechanisms underlying the relationship between social anxiety and smartphone addiction. This study explores the specific mechanisms underlying the relationship between social anxiety and smartphone addiction by examining the mediating role of rumination and the moderating role of gender. **Methods:** In five universities, 10 classes of university students were randomly selected from each university. A total of 2500 university students participated in the actual assessment throughout this study and completed the Mobile Phone Addiction Index Scale, Ruminative Responses Scale, and Interaction Anxiousness Scale. The data were analysed using SPSS 27.0. **Results:** The results of this study revealed that (1) rumination plays a partial mediating role in the relationship between social anxiety and smartphone addiction and that social anxiety not only has a direct effect on smartphone addiction but also indirectly affects smartphone addiction through rumination. (2) The mediating role of rumination between social anxiety and smartphone addiction is moderated by gender. **Conclusions:** Social anxiety is significantly and positively associated with smartphone addiction among university students, with rumination influencing this association. In addition, the indirect relationship between rumination and smartphone addiction was moderated by gender; however, the direct relationship between social anxiety and smartphone addiction was not affected by gender.

## 1. Introduction

Owing to developments in science and technology, the internet has infiltrated all facets of human activity. As one of the most important ways for individuals to access the internet, using smartphones brings great convenience to human life but also generates many psychological and behavioural problems. Excessive smartphone use can lead to serious consequences, especially smartphone addiction (SPA) [1]. Smartphone addiction is characterised as a behavioural addiction marked by obsessive smartphone use that results in different types of physical, psychological, or social detriment [2]. According to a previous report, university students constitute the main group of smartphone addicts, with a prevalence rate of more than 23.5% [3]. Smartphone addiction has also become a common problem among university students, and the abuse of mobile phones has adversely affected the physical and mental health of many students. This problem has received attention from all walks of life, so it is necessary to analyse the causes and mechanisms of university students’ smartphone addiction.

Research has shown that psychological variables such as stress, anxiety, self-regulation, and depression [4,5,6] are associated with smartphone addiction. Additionally, addiction to smartphones has a detrimental effect on an individual’s general health, academic performance, and social connections [7] and even increases the incidence of cyberbullying and ‘phubbing’ [8]. According to researchers, there is a connection between social anxiety and addiction to smartphones [9]. Social anxiety is one of the most direct contributors to smartphone addiction [10]. For instance, Brand et al. [11] suggested that people with symptoms of social anxiety will derive gratification by using the internet or mobile phone apps in lieu of offline interpersonal interactions and that these people expect online apps to help distract them from conflict or to free them from negative emotions.

In recent years, the increasing incidence of smartphone addiction among university students has garnered significant attention regarding the underlying factors contributing to this phenomenon. While social anxiety may pose a risk for smartphone addiction among university students, existing studies have inadequately explored the specific mechanisms linking social anxiety to smartphone addiction. This study seeks to investigate the mechanism by which social anxiety influences smartphone addiction through the development of a moderated mediation model. On this basis, promoting better mental health development in university students may have more obvious prevention and intervention effects.

### 1.1. The Relationship Between Social Anxiety and Smartphone Addiction

Social anxiety is anxiety that arises in real or imagined social situations out of an individual’s preconceived notions of other people’s evaluations or evaluations that are real [12]. Among the studies related to smartphone addiction, the I-PACE model (Person Affect Cognition Execution Model) of internet addiction proposed by Brand et al. [11] is more widely cited and is a process model that emphasises the fact that several core traits of a person, including personality and physiological factors, interacting with cognitive, affective and executive processes, can significantly predict an individual’s smartphone addiction status. Evidence indicates a correlation between media addiction and maladaptive psychological qualities, especially socialisation, namely, social anxiety [13].

Kardefelt-Winther’s [14] CIUT theory seeks to clarify the negative pressures and life events that compel individuals to engage in overreliance on technology to mitigate adverse emotions. In the CIUT, smartphone addiction is highlighted as a consequence of adverse life experiences; compensatory practices seek to mitigate stressor-induced unpleasant feelings, including anxiety. For example, people with high levels of social anxiety may compensate for feelings of loneliness by being on games or social apps because the online environment makes the individual feel safer, owing to anonymity [15]. This theory posits that excessive internet use functions as an avoidant coping mechanism employed by individuals to evade and alleviate undesirable feelings and consequences.

Many studies have shown that social anxiety is one of the top triggers of smartphone addiction [16,17]. Social anxiety among university students is currently as high as 45.7% [18]. People who suffer from social anxiety mitigate their face-to-face apprehension by engaging excessively in online socialisation, including the utilisation of smartphones [19]. Some studies on Chinese university students have shown that social anxiety may contribute to the development of smartphone addiction [13,20,21]. There are a number of cross-sectional studies and longitudinal studies with findings indicating that social anxiety has a significant effect on smartphone addiction and this relationship through mechanisms such as escapism [9] and emotion regulation [20]. At the same time, several meta-analyses and review articles have demonstrated this relationship [22,23,24]. However, some studies did not find a significant relationship between social anxiety and smartphone addiction, or the results were influenced by other variables (e.g., cultural background, sample characteristics [25], motivation to use [26]). According to a longitudinal study by Rozgonjuk et al. [27], social anxiety severity was not related to minutes of screen time but was negatively related to the frequency of unlocking the smartphone screen. This may be because people with higher levels of anxiety may be socially isolated or behaviourally avoidant [28], and, as a result, they are not motivated to check their mobile phones for emails, status updates from others, or notifications from social media. Additionally, research has shown that social anxiety is a positive predictor of problematic usage of social media [29], and, with the popularity of smartphones as mobile vehicles for social media, these studies can clearly extrapolate the association with smartphone addiction as well. Based on the existing research background, we propose the following hypothesis:

**Hypothesis** **1:** *Social anxiety positively predicts smartphone among Chinese university students.*

### 1.2. The Mediating Role of Rumination

A review of current studies indicates that rumination is a significant cognitive characteristic. Rumination is a negative cognitive style in which an individual experiencing a negative life event or negative emotional experience tends to use a negative response style to compulsively focus on and repeatedly think about the details of why the negative life event or negative emotional experience has occurred and what has resulted from it, which results in the individual experiencing more negative feelings [30]. Rumination activation maintains, exacerbates, or amplifies the vicious cycle between negative emotional experiences and negative cognitions. Empirical studies have shown that altered cognition in individuals caused by stress is an important reason that individuals can further exacerbate or amplify the transformation of negative emotions into internalised symptoms, such as anxiety and depression, after stress [31,32].

Research indicates that rumination encourages individuals to employ negative techniques for interpreting stressful circumstances, resulting in diminished problem-solving abilities [33]. The cognitive–behavioural model posits that unpleasant life experiences or stressors are remote and essential for the emergence of dysfunctional internet usage, whereas non-adaptive cognitions (rumination) are proximal for the pathological use of the internet to arise, with distal factors acting through proximal factors [34]. Mok et al. [35] argued that internet use and smartphone addictive use share many similarities and that both fall under the umbrella of behavioural addictions; thus, many studies have extended the application of this theoretical model to the field of smartphone addiction [10].

Studies indicate that increased levels of rumination correlate with heightened smartphone-addicted behaviour [36]. According to the I-PACE theoretical model proposed by Brand et al. [11], owing to erroneous flawed emotional and cognitive biases, people seek mobile phones to obtain transient pleasurable behavioural feedback to alleviate or escape negative emotions, and rumination, as one of the individual’s psycho-pathological factors, exacerbates erroneous behavioural responses and ultimately predisposes people to the occurrence of smartphone addiction. The I-PACE model further indicates that rumination may act as an indirect effect, such as mediation or moderation [37]. In addition to the theoretical support mentioned above, the present study proposes that the mediating role of rumination in the relationship between social anxiety and smartphone addiction is also grounded in relevant empirical research. Studies on rumination and smartphone addiction have shown that the greater the level of rumination an individual has, the greater their mobile phone addiction behaviour [36,38]. In addition, rumination can mediate the relationship between social anxiety and smartphone addiction among university students [10]. Considering that social anxiety might result in rumination, which subsequently contributes to smartphone addiction, a secondary hypothesis is suggested here:

**Hypothesis** **2:** *Rumination serves as a mediator in the association between social anxiety and smartphone addiction.*

### 1.3. The Moderating Role of Gender

Researchers have identified gender as a significant demographic variable in smartphone addiction. The findings suggest that women exhibit greater dependence on smartphones than men do, and this gender disparity intensifies with age [39]. Gender disparities are shown in several individual factors. Numerous studies have indicated that smartphone addiction differs by gender; for example, one study classified smartphone users into three groups, identifying women as having a greater propensity for developing smartphone addiction than males [40]. Mok et al. [35] reported that female university students had a greater propensity for smartphone overuse than their male counterparts did. Gender disparities are also evident in unpleasant emotions. Compared with male students, female students at universities reported significantly higher levels of anxiety on average [41]. Nevertheless, certain studies indicate that smartphone addiction does not vary substantially between genders [30]. Studies have shown that women are often more likely than men to develop smartphone addictive behaviours as a result of social anxiety [42,43]. However, other studies have shown that although girls report higher levels of social anxiety, boys show a stronger association between smartphone addiction and social anxiety symptoms [44]. The underlying reason for the gender difference may be that females are more likely to seek emotional support through their mobile phones, whereas males use them more for entertainment and information access [45,46].

On the basis of the aforementioned studies, the influence of gender on the three variables of smartphone addiction, rumination, and social anxiety cannot be overlooked. Although past studies have demonstrated considerable disparities between genders in terms of individual factors, studies on how gender moderates the link between these variables are scarce. The purpose of this study is to determine whether gender acts as a moderator in the relationships among social anxiety, rumination, and smartphone addiction, either directly or indirectly. Therefore, we suggest a third hypothesis:

**Hypothesis** **3:** *Direct and indirect correlations between social anxiety and rumination and smartphone addiction vary by gender.*

**Figure 1:**
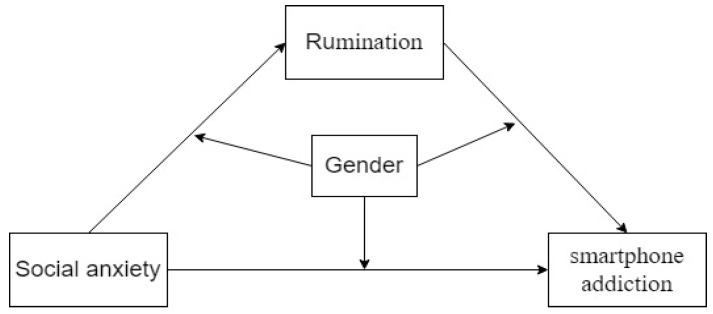
Framework of the theories in this study.

Figure 1 displays this study’s hypothesised model.

## 2. Methodology

### 2.1. Participants and Processes

This study takes university students in Zhengzhou City as the research subjects(subject). A multistage sampling technique was used to achieve this objective. An online survey was conducted from 20 December 2024 to 5 January 2025 to test smartphone addiction, rumination, and social anxiety among students at five institutions. The first stage is the selection of universities using random sampling. In the second stage, the study sample was selected on the basis of non-probability sampling techniques (i.e., purposive sampling). In this study, the recruitment of the respondents was based on the inclusion criteria. Briefly, they were selected on the basis of the following criteria: (1) Respondents had to be undergraduate students at any of the five universities selected. (2) Respondents must use a smartphone on a daily basis. Prior to the survey, online questionnaires were distributed and collected by faculty through our program ‘Questionnaire Star’ (https://www.wjx.cn/, 20 December 2024). The participants provided informed permission, and the objectives, utilisation, and importance of the data collection were elucidated, highlighting voluntary participation, anonymity, and secrecy. The participants possessed complete autonomy to terminate or withdraw from the questionnaire at any time. Approval was granted for this research project by the Ethics Committee of Universiti Sains Malaysia. (Approval number: USM/JEPeM/PP/24090798.)

A sample of 2500 students was selected for the online questionnaire survey. After fifteen days, a total of 2183 surveys were gathered, resulting in a recovery rate of 87.32%. Upon obtaining the data from the survey platform, they were then cleaned and organised to remove invalid questionnaires (incomplete responses and anomalous information that could not be processed) and to interpolate the means of each missing variable within a single sample. The total number of valid questionnaires for analysis was 1914, with a response rate of 76.56%. Among these, 937 were females (49%), and 977 were males (51%).

### 2.2. Measures

#### 2.2.1. Mobile Phone Addiction Index

The Mobile Phone Addiction Index was used to evaluate addiction to smartphones [39]. Using a total of seventeen questions, this assessment analyses four dimensions of smartphone addiction. These characteristics include the inability to control cravings, anxiety and disorientation, withdrawal/escapism, and decreased productivity. A five-point scale was used to evaluate the participants’ responses to these items. (1 = never, 5 = always). A greater total score signifies an elevated degree of smartphone addiction. The scale had a Cronbach’s α of 0.88 in this research.

#### 2.2.2. Ruminative Responses Scale

For the purpose of evaluating ruminative responses, the Chinese version of the Ruminative Responses Scale was used [30]. The RRS-10 includes two subscales (brooding and reflection), each with five items. Every item has a four-point Likert scale, ranging from “1” for “never” to “4” for “always”. Elevated scores signify an increased propensity for ruminative thinking. This study reported a Cronbach’s α of 0.93 for this scale.

#### 2.2.3. Interaction Anxiousness Scale

The Interaction Anxiousness Scale (IAS) was developed by Leary and Kowalski [47] to assess the propensity to experience anxiety in social situations, irrespective of patterns of inhibition, reticence, or avoidance. In this study, we used the Chinese version of the IAS translated by Ma et al. [48]. The scale has two dimensions, tension and relaxation, with 15 items, of which 4 are reverse-coded. The first dimension concerning stress comprises scores of 1, 2, 4, 5, 7, 8, 9, 11, 12, 13, and 14 as positive, whereas the subsequent dimension related to relaxation contains scores of 3, 6, 10, and 15 as negative. For the purpose of scoring, a five-point Likert scale is used, where a score of one equals “not at all characteristic of me”, and a score of five equals “extremely characteristic of me”. The scale has a Cronbach’s α of 0.87 in this study.

### 2.3. Data Processing and Analysis

All the statistical operations were performed using SPSS 27.0, which was used for the data analysis. First, we checked for common method bias using factor analysis. Second, SPSS 27.0 was used to conduct descriptive statistics, *t*-tests, chi-square tests, and Pearson correlation analyses. Afterwards, the mediating function of rumination in the connection between smartphone addiction and social anxiety was tested using Hayes’s PROCESS macro (Model 4) [49]. For ease of analysis, the independent, mediating and moderating variables were centred on the mean. Finally, Hayes’ PROCESS macro (Model 59) [50] was used to test the moderating role of gender in the mediation model.

## 3. Results

### 3.1. Preliminary Statistics

To investigate the probable bias of the conventional approach, a Harman single-factor test was carried out. A principal component analysis was carried out to examine each and every variable. A thorough investigation revealed that eight eigenvalues exceeded one. The initial factor accounted for 35.743% of the variance, falling short of the critical threshold of 40%. This finding suggests the absence of common method bias in the present research study [51].

For your convenience, the results of the descriptive statistics and the correlation analysis are shown in Table 1. The correlation analysis indicated that social anxiety was positively related to rumination (r = 0.561, *p* < 0.01) and smartphone addiction (r = 0.627, *p* < 0.01). There was a positive correlation between ruminating and addiction to smartphones (r = 0.594, *p* < 0.01). Furthermore, our study examined gender differences across research variables, as indicated by Table 2. Males had higher mean social anxiety scores than females, and, conversely, females scored significantly higher than males on smartphone addiction.

### 3.2. Testing for the Mediation Model

The SPSS macro model 4 developed by Hayes (2012) [49] was utilised to investigate the impact of rumination on the association between social anxiety and addiction caused by smartphones. The findings of the research suggested that social anxiety was a significant predictor in determining the likelihood of being addicted to a smartphone (β = 0.69, *p* < 0.001), even after controlling for gender and assuming that there was no mediating effect. After rumination was added as a mediating variable, social anxiety continued to be a substantial predictor of smartphone addiction (β = 0.46, *p* < 0.001). In addition, social anxiety was a significant predictor of rumination (β = 0.31, *p* < 0.001). Rumination was a significant predictor of smartphone addiction (β = 0.69, *p* < 0.001). As shown in Table 3.

The bias-corrected bootstrap tests also revealed that the direct influence factor was 0.46, with a 95% confidence interval ranging from 0.42 to 0.50. This figure represents 68.66% of the total influence. The indirect impact mediated by rumination was 0.21, with a 95% confidence range of 0.18–0.24, or 31.34% of the total effect. As shown in Table 4. The data suggest that rumination partially mediates the relationship between smartphone addiction and social anxiety.

### 3.3. Testing for the Moderated Mediation Model

In Hypothesis 3, we propose that gender is a moderator of the direct and indirect effects of social anxiety on rumination and smartphone addiction. This was done within the context of a mediation model that also included rumination and smartphone addiction. In this study, the proposed moderated mediation model was evaluated with the help of Hayes’s PROCESS macro (Model 59). The mediating variable in this study was rumination, and the moderating variable was gender. Hypothesis 2 has three separate subhypotheses: (a) gender influences the correlation between social anxiety and rumination; (b) gender influences the correlation between social anxiety and smartphone addiction; and (c) gender moderates the relationship between rumination and smartphone addiction. If hypothesis c is shown to be correct, this gives evidence that a moderated mediation model is in existence.

**Figure 2:**
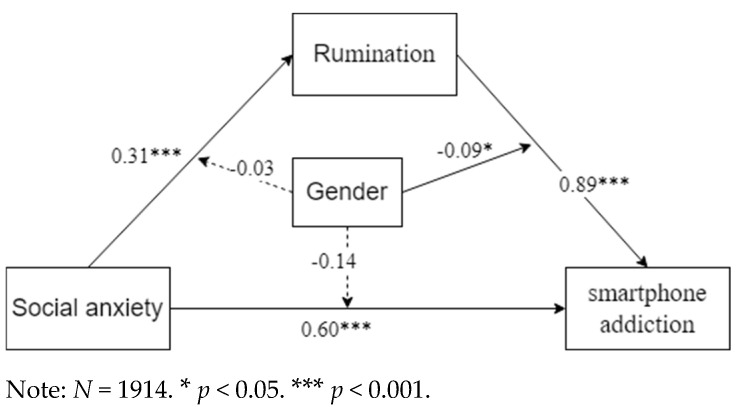
The moderated mediation pathways.

Table 5 indicates that gender did not regulate the direct pathway; instead, it affected the latter portion of the mediated pathway. The relationship between social anxiety and gender did not significantly influence rumination (β = −0.03, *p* > 0.05). The relationship between social anxiety and gender did not significantly influence smartphone addiction (β = −0.14, *p* > 0.05). The interplay between rumination and gender significantly influenced smartphone addiction (β = −0.09, *p* < 0.05). This suggests that gender plays a role in mitigating the relationship between rumination and smartphone addiction, thus providing support for hypothesis c. Thus, the results indicate partial support for Hypothesis 2. These data indicate that gender influences the relationship between rumination and smartphone addiction. Figure 2 illustrates the moderated mediator route along with the associated path weights.

**Figure 3:**
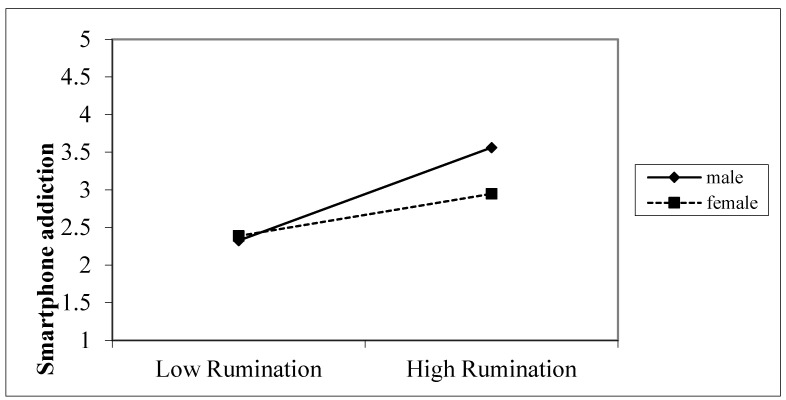
Relationship between rumination and smartphone addiction.

The moderating influence of gender on the relationship between rumination and smartphone addiction was investigated via simple slope analyses. A simple slope plot of rumination versus smartphone addiction was developed, as shown in Figure 3. In the male cohort, rumination was a significant predictor of smartphone addiction (simple slope = 0.623, t = 13.931, *p* < 0.001). Conversely, in the female cohort, the correlation between rumination and smartphone addiction was less pronounced (simple slope = 0.179, t = 1.634, *p* = 0.104). This finding indicates that the male cohort exhibited heightened smartphone addiction as ruminating intensified.

## 4. Discussion

This study utilised the I-PACE model [11] and Kardefelt-Winther’s [14] Compensatory Use Theory (CIUT) to develop a moderated mediation model for investigating the correlation between social anxiety and addiction to smartphones. The model investigates the role that rumination plays as a mediator in the connection between social anxiety and smartphone addiction among university students while simultaneously taking into account the moderating effect that gender has on this mediation. The results of this study demonstrate that rumination regulates the connection between social anxiety and smartphone addiction. The difference in gender was found to affect the correlation between rumination and addiction to smartphones.

### 4.1. Social Anxiety and Smartphone Addiction

There was a significant positive association between social anxiety and smartphone addiction among university students. According to the findings of the present study, there was a correlation between the intensity of social anxiety and the intensity of smartphone addiction, which is consistent with the findings of other studies [13]. The main characteristic of social anxiety is the fear of judgment from others, where the individual believes that other people will give negative feedback, which leads to fear when communicating [52]. In the new social communication model, college students can avoid face-to-face communication, thus reducing the possibility of cognitive processing bias due to negative expressions from others [53]. As a result, students can be more comfortable communicating via mobile phones and the internet [54]. People with high levels of social anxiety tend to choose mobile phones or the internet as a substitute for real social interaction and ultimately show a high level of dependence on mobile phones and the internet. In addition, individuals with high social anxiety tend to have poorer emotion regulation skills, whereas smartphones themselves, in addition to social functions, are also associated with nonsocial functions, with a wealth of entertaining functional applications such as various games and video playback applications, which can be used as external tools for emotion regulation [55]. It can assist those with significant social anxiety in combating numerous negative emotions; nevertheless, it may also facilitate excessive reliance on smartphones.

### 4.2. The Mediating Influence of Rumination

Rumination was found to play a role in mediating the connection between social anxiety and addiction to smartphones, according to the findings of this research. The conclusions of the present study align with findings from another related study [10]. Rumination causes people to focus their attention primarily on thinking about negative emotions repeatedly, becoming deeply involved in them and unable to extricate themselves. This not only exacerbates the severity of people’s psycho-spiritual disorders but can also inhibit their ability to recover from bad moods [56]. Moreover, the I-PACE model [11] proposes the presence of intermediate variables, particularly affective and cognitive response variables. These variables play a significant role in elucidating the connection between susceptibility and smartphone addiction. Some examples of these variables include cognitive and attentional biases, coping strategies, inhibitory control, and craving. In addition, rumination is often considered an affective and cognitive bias that drives smartphone addiction. The emergence of ruminative thinking triggers ego depletion and excessive use of cognitive resources, resulting in individuals not having sufficient cognitive resources to cope with stressful events, a negative cognitive processing style that is a common core element in the emergence of social anxiety and smartphone addiction [57]. This suggests that rumination is a cognitive risk factor that exacerbates the vicious cycle between negative emotions and negative cognitions [58]. It is a catalyst for the development of smartphone addictive behaviours as a result of social anxiety. Thus, rumination can serve as a mediating variable between anxiety-related psychiatric disorders and the severity of smartphone addiction [37].

### 4.3. The Moderating Influence of Gender

Further research confirmed that gender plays a moderating role in the second half of the mediating pathway. Specifically, while rumination was significantly associated with smartphone addiction among university students, this effect was more pronounced among males. This is due to social role theory [59], which suggests that society expects different roles from men and women, with male students being expected to ‘fight’ and female students being expected to ‘run away’ from their roles. Society has a lower tolerance for men’s expression of negative emotions, and they are prone to suppress or control their own responses to their own thinking patterns. Consequently, boys exhibiting elevated rumination are inclined to incessantly contemplate the origins and possible repercussions of adverse events, thereby exacerbating the influence of risk variables in precipitating smartphone addiction. Unlike females, who tend to seek support from social relationships [60], they may cope with stress by playing mobile games or watching short videos. Therefore, males are more likely to suffer from smartphone addiction than females.

This study examined the intrinsic mechanism between social anxiety and smartphone addiction in university students, which is important for the prevention and treatment of smartphone addiction in university students. Schools can start from the perspective of changing university students‘ negative cognitive styles and by adjusting individuals’ negative thinking patterns, thereby buffering or partially offsetting the negative impact of social anxiety on university students’ smartphone addiction. Specifically, schools can enrich the extracurricular life of college students by organising colourful extracurricular quality activities (e.g., campus singing contests, reading activities, etc.) to divert the attention of individuals from their social anxiety to reduce students’ social anxiety. In addition, through the intervention of rumination (e.g., positive mindfulness therapy [61], group counselling [62]), university students can be guided to gradually adjust their cognitive style through activities. Finally, gender differences should be taken into account in designing interventions.

## 5. Limitations and Future Directions

This study established a mediation model with moderating effects to examine the links among the variables. There are several shortcomings in this study due to its limitations. Firstly, the cross-sectional design self-report questionnaire method used in this study does not allow for the argumentation of relationships between variables. Therefore, more investigations that are either longitudinal or experimental are needed to validate the causal correlations that were found in this research. Second, the functions that various varieties of smartphone addiction (such as mobile game addiction and mobile media addiction) play in mitigating or regulating the relationship between the two have not been studied. Because this is the case, it is strongly suggested that any future empirical research investigating the connection between social anxiety and addiction to smartphones concentrate more on particular types of addiction to smartphones. Finally, other demographic variables were not considered in this study, and, in future studies, diverse demographic characteristics should be analysed, such as region, age, and grade level.

In conclusion, this study demonstrated that university students’ social anxiety can influence smartphone addiction through rumination and that gender has a moderating effect on rumination’s influence on smartphone addiction. However, other mediating or moderating variables in the process by which social anxiety affects smartphone addiction may exist, which could not be examined in detail.

## 6. Conclusions

In summary, this research concluded that social anxiety is significantly and positively associated with smartphone addiction among university students, with rumination potentially influencing this association. In addition, the indirect relationship between rumination and smartphone addiction was moderated by gender; however, the direct relationship between social anxiety and smartphone addiction was not affected by gender. Furthermore, the association between rumination and smartphone addiction was more pronounced among male university students than among their female counterparts.

## Figures and Tables

**Table 1:** Descriptive statistics and correlations for all variables.

Variables	M	SD	1	2	3	4
1. Social anxiety	46.80	14.88	1			
2. Rumination	24.70	8.13	0.561 **	1		
3. Smartphone addiction	49.05	15.94	0.627 **	0.594 **	1	

Note: *N* = 1914. ** *p* < 0.01.

**Table 2:** Gender differences in variables.

Variables	Full Example	Male	Female	*p*
	M ± SD	M ± SD	M ± SD	
Social anxiety	46.80 ± 14.88	47.86 ± 14.88	45.74 ± 15.19	0.001 **
Rumination	24.70 ± 8.13	24.38 ± 8.19	25.03 ± 8.04	0.057
Smartphone addiction	49.05 ± 15.94	48.46 ± 15.50	49.67 ± 16.37	0.003 **

** *p* < 0.01.

**Table 3:** The mediation model of rumination in the relationship between social anxiety and smartphone addiction.

Predictors	Rumination			Smartphone Addiction		
	β	SE	95% CI	β	SE	95% CI
Social anxiety	0.31 ***	0.01	[0.28, 0.32]	0.46 ***	0.02	[0.42, 0.50]
Rumination				0.69 ***	0.04	[0.61, 0.77]

Note. *N* = 1914. *** *p* < 0.001

**Table 4:** Bootstrapping of the indirect impact and the 95% confidence interval (CI) for the mediation model.

	Estimated Effect	SE	95% CI	Ratio to Total Effect
Total effect	0.67	0.02	[0.63, 0.71]	
Direct effect	0.46	0.02	[0.42, 0.50]	68.66%
Indirect effect	0.21	0.01	[0.18, 0.24]	31.34%

Note. *N* = 1914.

**Table 5:** Effect of moderated mediation between smartphone addiction and social anxiety.

Predictors	Rumination			Smartphone Addiction		
	β	SE	95% CI	β	SE	95% CI
Social anxiety	0.31 ***	0.01	[0.29, 0.33]	0.60 ***	0.07	[0.46, 0.73]
Rumination				0.89 ***	0.12	[0.65, 1.13]
Gender	0.68 *	0.31	[0.08,1.28]	0.82 ***	0.53	[0.21, 0.82]
Social anxiety * × Gender	−0.03	0.02	[−0.07,0.01]	−0.14	0.08	[−0.29, 0.01]
Rumination × Gender				−0.09 *	0.04	[−0.17, −0.01]
*R* ^2^	0.32			0.48		
*F*	87.59 ***			35.79 ***		

Note: *N* = 1914. * *p* < 0.05. *** *p* < 0.001.

## Data Availability

The datasets used and/or analysed during the current study are available from the author upon reasonable request.
